# Epidemiology and Natural History of Human Papillomavirus Infections in the Female Genital Tract

**DOI:** 10.1155/IDOG/2006/40470

**Published:** 2006-01-30

**Authors:** Kevin A. Ault

**Affiliations:** Department of Gynecology and Obstetrics, Emory University School of Medicine, Atlanta, GA 30303, USA

## Abstract

Human papillomavirus (HPV) is the most common newly diagnosed sexually transmitted infection in the United States. Although the majority of sexually active adults will be infected with HPV at least once in their lives, it is sexually active women less than 25 years of age who consistently have the highest rates of infection. Besides youth and gender, common risk factors for HPV infection and clinical sequelae of infection include high number of sexual partners and coinfection with *Chlamydia trachomatis* or herpes simplex virus. Most HPV infections are cleared by the immune system and do not result in clinical complications. Clinical sequelae in cases of low-risk HPV infection consist of genital warts, and clinical manifestations of high-risk HPV infection include abnormal Pap test results, low-grade squamous intraepithelial lesions (LSIL), high-grade squamous intraepithelial lesions (HSIL), and cervical cancer. LSIL, HSIL, and cervical cancer carry significant morbidity and/or mortality; genital warts and abnormal Pap test results are often significant sources of psychosocial distress. Currently, there are neither effective means of preventing HPV transmission nor cures for clinical manifestations: infection can only be prevented via complete sexual abstinence, while treatment for clinical sequelae such as genital warts and cytologic abnormalities consists of removing the problematic cells and watching for recurrence; this method consumes significant health care resources and is costly. New prophylactic HPV vaccines promise to dramatically reduce the incidence of HPV infection, genital warts, and cytologic abnormalities.

## INTRODUCTION

Human papillomavirus (HPV) is a significant source of morbidity and mortality in the United States and worldwide. High-risk, oncogenic HPV types (including HPV 16 and HPV 18) are associated with 99.7% of all cervical cancers, as well as low-grade squamous intraepithelial lesions (LSIL), high-grade squamous intraepithelial lesions (HSIL), and abnormal Papanicolaou (Pap) test results, which carry significant health care costs and psychosocial morbidity. Low-risk HPV types (HPV 6 and HPV 11) are responsible for additional abnormal Pap test results, as well as almost all cases of genital warts. HPV is so common that more than half of all sexually active adults will be infected in their lifetime, although young, sexually active women bear the brunt of both infection and clinical complications. Currently there are neither effective HPV prevention strategies nor good treatments for individuals with genital warts or cervical lesions; available treatments focus on removing the affected area, and recurrence is common. Prophylactic vaccines will soon become available, and promise to significantly reduce the morbidity and mortality associated with these infections.

## EPIDEMIOLOGY OF HUMAN PAPILLOMAVIRUS INFECTIONS

**Figure 1:**
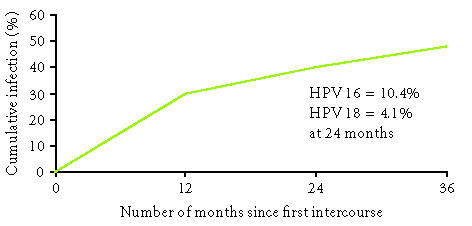
Cumulative rate of HPV infection among college-aged women who were virgins at baseline. Adapted from Winer et al [3].

Approximately 6.2 million new HPV infections occur every year in the United States, and approximately 20 million individuals are currently infected [1]. HPV is spread by skin-to-skin sexual contact and is prevalent in all sexually active populations. The Centers for Disease Control estimates that at least half of all sexually active individuals will acquire HPV at some point in their lives, whereas at least 80% of women will acquire an HPV infection by age 50 [1]. In the United States, it is estimated that 10% of the population has an active HPV infection, 4% has an infection that has caused cytological abnormalities, and an additional 1% have infection causing genital warts [2]. Although 1% of Americans have clinically visible genital warts, as many as 13% of those attending STD clinics have genital warts [2]. The greatest risk factors for infection are gender, youth, and sexual activity, with the highest rates being consistently found in sexually active women less than 25 years of age. Winer et al followed 148 female university students as they initiated sexual activity (Figure 1) [3]. They found a cumulative incidence of HPV of 38.9% at 24 months. HPV 16 was the most common type, with a cumulative infection rate of 10.4% at 24 months; the cumulative incidence of HPV 18 infection was 4.1% for the same time period. Brown et al studied a smaller cohort of mid-adolescent women for two years. Of the women studied, 82% were infected with HPV during the 2-year study period [4]. DNA from both low-risk and high-risk HPV types has even been found in women who have sex with women, a population that would be expected to have a low incidence of HPV infection [5]. It should be noted that prevalence estimates vary depending on the technique used to assess viral load; polymerase chain reaction analysis is a more sensitive detection method and yields higher rates of prevalence.

**Figure 2:**
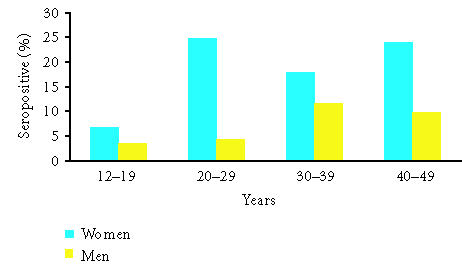
Seroprevalence of HPV 16 by age and gender. Modified from Stone et al [6].

HPV 16 alone is linked to more than 50% of all cervical cancers [6]; thus, the prevalence of HPV 16 is of special interest. One study utilized an experimental serological test to determine the presence of antibodies to HPV 16, which signify prior exposure to HPV, instead of the more commonly assessed viral DNA which is indicative of active infection. More than 7000 sera were tested from a national sample from the United States. Gender and age specific findings are shown in Figure 2. Women were more likely to be seropositive for HPV 16 (17.9%) than were men (7.9%). However, this methodology may in fact underestimate the true prior exposure to HPV 16, because <60% of women infected with HPV 16 develop type-specific antibodies [6].

Sexual activity is the primary risk factor for HPV infection, but condoms, although effective at preventing the spread of many other sexually transmitted infections, may not prevent all HPV infections. A meta-analysis of more than 20 trials investigating the role of condoms in HPV transmission and the development of clinical complications concluded that, although condoms do not protect against cervical infection, they may offer some protection against HPV-associated disease. Specifically, although there is conflicting evidence as to whether condom use protects against CIN 2/3, condoms may protect against cervical cancer [7]. A recent prospective study by Hogewoning et al studied the effect of condom use on the regression of CIN lesions. Women with abnormal cervical smears or CIN were randomized to use condoms after the initial diagnosis. The 2-year cumulative regression rate was 53% in the “condom” group and 35% in the “noncondom” group. The 2-year cumulative rate of HPV clearance was 23% in the condom group and 4% in the noncondom group [8]. It is difficult to accurately assess the role of condoms in preventing HPV infection and the development of clinical complications of infections, not least because investigators rely on patient self-report to assess condom use. The available evidence does, however, suggest that condom use protects against some clinical sequelae of HPV infection and aids clearance of infection and clinical symptoms, even if it does not prevent primary infection.

## NATURAL HISTORY OF HPV INFECTION

HPV is a small DNA virus with a genome of approximately 8000 base pairs [9]. HPV targets the basal cells in the stratified squamous epithelium and the metaplastic cells at the squamocolumnar junction of the cervix. Additionally, HPV may infect the glandular epithelium of the endocervix, resulting in glandular neoplasms, such as adenocarcinoma in situ or invasive adenocarcinoma [10]. The two primary oncogenes of high-risk HPV types are E6 and E7. The “E” designation indicates that these two genes are expressed early in the HPV life cycle. The products of these two genes alter host-cell metabolism to favor neoplastic development. E6 binds to and degrades the host-cell protein p53. An effect of this targeted degradation is to prevent apoptosis of the infected host epithelial cells. Telomerase is also activated, further augmenting oncogenic changes. The E7 protein has a similar effect on cell metabolism by binding to retinoblastoma protein, inhibiting its function. This leads to disruption of the cell cycle [9]. In addition, E6 and E7 proteins may cause chromosomal destabilization, and inhibit cyclin-dependent kinase inhibitors and host interferons [11].

The degree of expression of HPV E6 and E7 is highly correlated with the type of cervical lesion: in low-grade lesions, E6 and E7 are expressed at low levels in the basal cells and higher levels in the upper layers of the epithelium, whereas in high-grade lesions E6 and E7 are expressed at high levels throughout the epithelium [9]. In low-grade lesions, HPV is in episomal form, whereas in higher grade lesions and cancer, the HPV DNA is more likely to have been integrated into the host-cell chromosome. The integration of HPV DNA into the host DNA increases cellular proliferation and the chance of malignancy [9].

HPV infection, unlike many genitourinary infections, is not usually associated with immediate symptoms such as itching, burning, and vaginal discharge [12]. Rather, the majority of those infected with HPV will not develop clinical disease or symptoms because the host immune system resolves most infections. In one study, only 24.8% of women infected with HPV 6 or 11 actually developed genital warts [12]. A large, prospective 10-year cohort study of more than 20,000 women enrolled in a health maintenance organization found that the incidence of CIN 3 or cancer was approximately 7% in HPV-positive women for the duration of the study [13]. Thus, only the minority of patients with HPV infections develop serious clinical complications. The exact mechanism by which HPV infection is cleared by the host immune system is currently unknown.

A number of factors are associated with an increased risk of initial infection and/or clinical sequelae such as genital warts, CIN or invasive cancer. Individuals who smoke are more likely to develop cancer, and it is thought that smoking increases the likelihood of developing SIL [14]. Herpes simplex virus (HSV) and *C. trachomatis* infection are also associated with cervical cancer. Smith et al performed a pooled analysis that found prior exposure to HSV-2 was associated with 2-fold increased risk of squamous cell carcinoma of the cervix in patients with HPV [15]; *C. trachomatis* infection is associated with a similarly increased risk of squamous cell carcinoma [16]. *C. trachomatis* infection is also associated with more persistent HPV infection, which may contribute to the increased risk of clinical complications of HPV infection in individuals coinfected with *C. trachomatis* [17].

Immunosuppressed and HIV positive individuals are at a high risk of both HPV infection and HPV-associated disease. High HIV RNA levels and CD4 < 200 cells per mm^3^ counts are associated with both incident and persistent HPV infection, although the association with incident infection is stronger [18]. Among women with oncogenic HPV, HIV-positive women with low CD4 cell counts are more likely than either HIV-negative women or HIV-positive women with high CD4 cell counts to develop SIL [19]. Data concerning the effect of highly active anti-retroviral therapy on the natural history of HPV infections and cervical dysplasia are mixed and further investigation is needed in this area.

## HPV INFECTION AND CERVICAL CANCER

**Figure 3:**
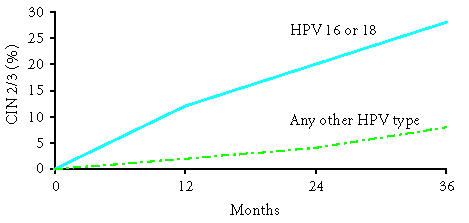
Cumulative risk of CIN 2/3 after infection with HPV 16 or 18, or other types. Adapted from Winer et al [20].

Steps that occur from initial infection leading to the development of cancer include overcoming host immune resistance, possible integration of HPV DNA into the host genome, and accumulation of additional mutations within the infected host cell [11]. HPV must be persistent within the host epithelial cells as a preliminary step toward advanced neoplastic changes. The traditional view has been that this process takes years, if not decades, to occur after initial HPV infection. Recent studies suggest that these changes may develop more quickly than previously thought. Winer et al followed women after initial HPV infection for the development of CIN 2/3. As shown in Figure 3, approximately 27% of women with an initial HPV 16 or 18 infection progressed to CIN 2/3 within 36 months [20]. A second study of a large health maintenance cohort found that approximately 20% of women 30 years of age or older who were initially infected with HPV 16 developed CIN 3 or cervical cancer within 120 months. Women who had an initial HPV 18 infection had approximately a 15% risk of developing CIN 3 or cervical cancer at 120 months [21].

The strong correlation between infection with high-risk types of HPV and LSIL, HSIL, and cervical cancer suggests that HPV DNA testing would be a useful tool for the management of women with abnormal Pap test results, especially in the case of those with equivocal test results. In the case of an equivocal Pap test result, HPV DNA testing can help determine whether the individual should be referred for colposcopic assessment [22].

## COSTS OF INFECTION

HPV is one of the most common infections of the female genital tract, and it is also one of the most costly. HPV-associated health care costs include routine Pap tests, treatment of genital warts, follow-up of cytological abnormalities, and management of cervical malignancies. An observational study of the Kaiser Permanente health plan found that the annual cost of screening and treatment of HPV-related cervical neoplasia was $26,415 per 1000 women, with nearly 2/3 of that ($16,746) attributable to routine screening [23]. Another large study investigated the incidence and costs associated with genital warts: each episode of genital warts required an average of 3.1 doctor visits, with an average total cost of $436 for all visits. Incidence ranged from 1.7 cases per 1000 patient-years overall to a peak of 6.2 cases of genital warts per 1000 person-years, in women aged 20 to 24 years, at a cost of $1692 per 1000 person-years, and 5 cases per 1000 patient-years in men aged 25 to 29 at a cost of $1717 per 1000 patient-years [24].

Currently there are no good methods for preventing HPV infection. Nor are there comprehensive and effective treatments for the clinical consequences of infection: for both genital warts and lesions, management entails removal of discrete lesions and monitoring for recurrence. Prophylactic HPV vaccines that confer protection against both high- and low-risk HPV types are expected to substantially reduce the burden of HPV-associated disease. A bivalent vaccine formulated to protect against the two most common high-risk HPV types, 16 and 18, and a quadrivalent vaccine that will protect against HPV 16 and 18, and the two most common low-risk types, HPV 6 and 11, are in ongoing clinical testing. Phase 2 data suggest that both vaccines are safe, are capable of producing immune responses in orders of magnitude larger than those produced in the wake of a natural infection, and are more than 90% effective at preventing persistent HPV infection and the clinical manifestations of HPV infection; recent phase 3 data suggest that the quadrivalent vaccine may be 100% effective at preventing HPV 16/18-associated disease [25–27].

## CONCLUSION

The vast majority of sexually active men and women will become infected with HPV. Although the host immune system successfully clears most of these infections, some women will develop fulminant disease such as genital warts, cervical dysplasia, and invasive cervical cancer. Current treatment options are not curative; therefore, preventative vaccines that lower the incidence of HPV infection and its associated diseases may offer a promising alternative to current therapies.

## References

[1] Centers for Disease Control and Prevention (2004). Genital HPV Infection—CDC Fact Sheet. Centers for Disease Control and Prevention.

[2] Koutsky LA (1997). Epidemiology of genital human papillomavirus infection. *The American Journal of Medicine*.

[3] Winer RL, Lee SK, Hughes JP, Adam DE, Kiviat NB, Koutsky LA (2003). Genital human papillomavirus infection: incidence and risk factors in a cohort of female university students. *American Journal of Epidemiology*.

[4] Brown DR, Shew ML, Qadadri B (2005). A longitudinal study of genital human papillomavirus infection in a cohort of closely followed adolescent women. *The Journal of Infectious Diseases*.

[5] Marrazzo JM, Koutsky LA, Kiviat NB, Kuypers JM, Stine K (2001). Papanicolaou test screening and prevalence of genital human papillomavirus among women who have sex with women. *American Journal of Public Health*.

[6] Stone KM, Karem KL, Sternberg MR (2002). Seroprevalence of human papillomavirus type 16 infection in the United States. *The Journal of Infectious Diseases*.

[7] Manhart LE, Koutsky LA (2002). Do condoms prevent genital HPV infection, external genital warts, or cervical neoplasia? A meta-analysis. *Sexually Transmitted Diseases*.

[8] Hogewoning CJ, Bleeker MC, van den Brule AJ (2003). Condom use promotes regression of cervical intraepithelial neoplasia and clearance of human papillomavirus: a randomized clinical trial. *International Journal of Cancer*.

[9] Scheurer ME, Tortolero-Luna G, Adler-Storthz K (2005). Human papillomavirus infection: biology, epidemiology, and prevention. *International Journal of Gynecological Cancer*.

[10] Longworth MS, Laimins LA (2004). Pathogenesis of human papillomaviruses in differentiating epithelia. *Microbiology and Molecular Biology Reviews*.

[11] zur Hausen H (2000). Papillomaviruses causing cancer: evasion from host-cell control in early events in carcinogenesis. *Journal of the National Cancer Institute*.

[12] Mao C, Hughes JP, Kiviat NB (2003). Clinical findings among young women with genital human papillomavirus infection. *American Journal of Obstetrics and Gynecology*.

[13] Sherman ME, Lorincz AT, Scott DR (2003). Baseline cytology, human papillomavirus testing, and risk for cervical neoplasia: a 10-year cohort analysis. *Journal of the National Cancer Institute*.

[14] Castellsagué X, Muñoz N (2003). Chapter 3: Cofactors in human papillomavirus carcinogenesis—role of parity, oral contraceptives, and tobacco smoking. *Journal of the National Cancer Institute Monographs*.

[15] Smith JS, Herrero R, Bosetti C (2002). Herpes simplex virus-2 as a human papillomavirus cofactor in the etiology of invasive cervical cancer. *Journal of the National Cancer Institute*.

[16] Anttila T, Saikku P, Koskela P (2001). Serotypes of *Chlamydia trachomatis* and risk for development of cervical squamous cell carcinoma. *JAMA: The Journal of the American Medical Association*.

[17] Samoff E, Koumans EH, Markowitz LE (2005). Association of *Chlamydia trachomatis* with persistence of high-risk types of human papillomavirus in a cohort of female adolescents. *American Journal of Epidemiology*.

[18] Strickler HD, Burk RD, Fazzari M (2005). Natural history and possible reactivation of human papillomavirus in human immunodeficiency virus-positive women. *Journal of the National Cancer Institute*.

[19] Harris TG, Burk RD, Palefsky JM (2005). Incidence of cervical squamous intraepithelial lesions associated with HIV serostatus, CD4 cell counts, and human papillomavirus test results. *JAMA: The Journal of the American Medical Association*.

[20] Winer RL, Kiviat NB, Hughes JP (2005). Development and duration of human papillomavirus lesions, after initial infection. *The Journal of Infectious Diseases*.

[21] Khan MJ, Castle PE, Lorincz AT (2005). The elevated 10-year risk of cervical precancer and cancer in women with human papillomavirus (HPV) type 16 or 18 and the possible utility of type-specific HPV testing in clinical practice. *Journal of the National Cancer Institute*.

[22] Cox JT (2003). The clinician's view: role of human papillomavirus testing in the American Society for Colposcopy and Cervical Pathology Guidelines for the management of abnormal cervical cytology and cervical cancer precursors. *Archives of Pathology & Laboratory Medicine*.

[23] Insinga RP, Glass AG, Rush BB (2004). The health care costs of cervical human papillomavirus-related disease. *American Journal of Obstetrics and Gynecology*.

[24] Insinga RP, Dasbach EJ, Myers ER (2003). The health and economic burden of genital warts in a set of private health plans in the United States. *Clinical Infectious Diseases*.

[25] Harper DM, Franco EL, Wheeler C (2004). Efficacy of a bivalent L1 virus-like particle vaccine in prevention of infection with human papillomavirus types 16 and 18 in young women: a randomized controlled trial. *The Lancet*.

[26] Villa LL, Costa RLR, Petta CA (2005). Prophylactic quadrivalent human papillomavirus (types 6, 11, 16, and 18) L1 virus-like particle vaccine in young women: a randomised double-blind placebo-controlled multicentre phase 3 efficacy trial. *The Lancet Oncology*.

[27] Skjeldestad FE FUTURE II steering committee. Prophylactic Quadrivalent Human Papillomavirus (HPV) (Types 6, 11, 16, 18) L1 Virus-Like Particle (VLP) Vaccine (Gardasil) Reduces Cervical Intraepithelial Neoplasia (CIN) 2/3 Risk.

